# Online Color Classification System of Solid Wood Flooring Based on Characteristic Features

**DOI:** 10.3390/s21020336

**Published:** 2021-01-06

**Authors:** Zilong Zhuang, Ying Liu, Fenglong Ding, Zhengguang Wang

**Affiliations:** College of Mechanical and Electronic Engineering, Nanjing Forestry University, Nanjing 210037, China; zzl0702@njfu.edu.cn (Z.Z.); dfl@njfu.edu.cn (F.D.); nanlinwzg@njfu.edu.cn (Z.W.)

**Keywords:** wood color classification, XGBoost, color feature

## Abstract

Solid wood flooring has good esthetic properties and is an excellent material for interior decoration. To meet the artistic effects of specific interior decoration requirements, the color of solid wood flooring needs to be coordinated. Thus, the color of the produced solid wood flooring needs to be sorted to meet the individual needs of customers. In this work, machine vision, deep learning methods, and ensemble learning methods are introduced to reduce the cost of manual sorting and improve production efficiency. The color CCD camera was used to collect 108 solid wood floors of three color grades provided by the company and obtained 108 18,000 × 2048 pixel wood images. A total of 432 images were obtained after data expansion. Deep learning methods, such as VGG16, DenseNet121, and XGBoost, were compared. After using XGBoost to filter the features, the accuracy of solid wood flooring color classification was 97.22%, the training model time was 5.27 s, the average test time for each picture was 51 ms, and a good result was achieved.

## 1. Introduction

Solid wood flooring is made of natural wood. It has the functions of adjusting temperature and controlling air humidity. It has no pollution release during use. In addition, its natural texture and color can bring a strong sense of comfort, which is excellent for indoor decoration [1]. As early as the European Baroque art period, solid wood flooring has become a decorative material that wealthy people prefer to use. People process different colors of natural bark into artistic patterns that meet specific interior decoration needs to enhance decorative effect and grade. With the current increase in the demand for wood, improving the production efficiency and yield of solid wood flooring has become a requirement for flooring companies. The manual color sorting of solid wood floors has low production efficiency and high labor costs. Thus, the introduction of machine vision technology to sort the colors of solid wood floors can improve the automation and intelligence level of the solid wood floor production process and efficiency greatly.

Classification algorithms have always been a hotspot in data mining research [2]. The color classification and classification prediction of solid wood flooring are processes of mining feature information in image data.

At present, the color classification of wood usually begins from two directions: one is to extract better feature values, and the other is to select a more accurate classifier. Some studies have designed better color characteristics to describe the wood color [3,4,5,6]. Barmpoutis [7] proposed a method to enable the representation of wood images as concatenated histograms of higher-order linear dynamical systems produced by vertical and horizontal image patches; Hiremath [8] proposed an efficient multiresolution method for texture classification based on anisotropic diffusion and local directional binary patterns (LDBP). Some studies used more accurate classification algorithms for classification [9,10]. Nasir [11] compared and evaluated the performance of artificial neural networks (ANN), SVM, and naive Bayes (NB) classifiers for thermowood classification and obtained the conclusion that the SVM and NB models can be used for online quality control. Nasir [12] used the “adaptive neuro-fuzzy inference system” (ANFIS), “Group Method of Data Handling” (GMDH), and “multilayer perceptron” (MLP) neural networks for predicting the wood properties and found that the ANFIS and GMDH neural network are better than MLP. With the development of machine learning, deep learning methods have also been used in the non-destructive testing of solid wood [13]. Li [14] presented a split-shuffle-residual (SSR)-based CNN that can learn features automatically from wood images for real-time classification of rubber wood boards.

Many methods can be used for detecting and classifying wood surface colors using machine vision [15]. However, only few color classifications for the entire board are currently available. The SVM algorithm is sensitive to missing data, and there is no general solution to the nonlinear model; the convolutional neural network algorithm input image size is constrained, the required training set data are large, and the training time is long; and Bayesian classification needs to know the prior probability, and the classification error exists. The KNN algorithm is a lazy learning method with slow calculation speed, weak interpretability of the output, and difficulty in determining the K value. Meanwhile, K-means [16] is an unsupervised learning method. Like the KNN method, the K value is not easy to select, and a local optimal solution is obtained, which is sensitive to noise. The classification effect is greatly affected by the selection of the initial clustering center. XGBoost uses regularization to prevent overfitting, can customize the loss function, uses parallel optimization, and has high efficiency. It also supports column sampling, which can reduce overfitting and calculation. This paper extracted the color features of a wood surface and then used XGBoost [17] (eXtreme Gradient Boosting) for classification. Subsequently, the importance of each color feature value on the final classification result was calculated to screen for achieving the effect of using the least feature values to make the best prediction.

The innovations of this paper are as follows: (a) introducing the XGBoost classification model in the color classification of solid wood flooring; and (b) removing features with low variance to select the most important features in wood color classification. The contributions of this paper are (a) color classification of the whole piece of solid wood flooring; and (b) classification and screening of the importance of the color features of solid wood flooring and finding the most important color features to improve the speed and accuracy of color classification.

## 2. Materials and Methods

### 2.1. Imaging

**Figure 1:**
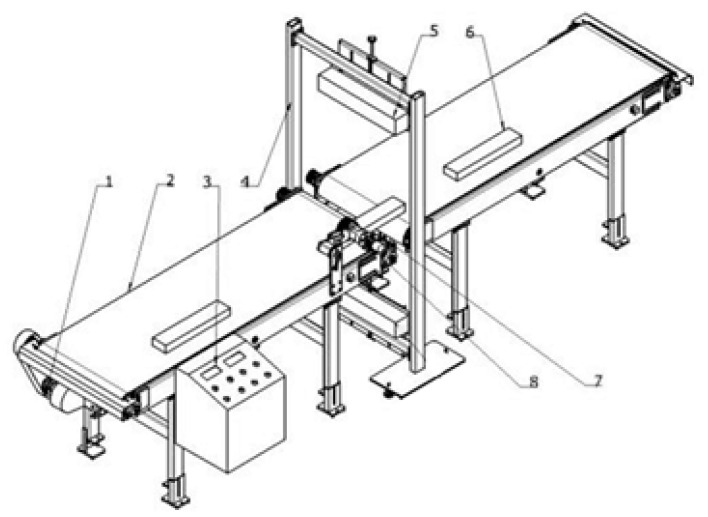
Image acquisition device. 1. Transmission device, 2. Transmission belt, 3. Control panel, 4. CCD camera holder, 5. CCD camera, 6. Sample solid wood flooring, 7. Photoelectric sensor, 8. Pressure wheel.

In the actual production, performing color classification on the image of the solid wood flooring processed through several processes is necessary. The structure of the image acquisition system built in this article is shown in Figure 1. The acquisition system mainly includes four components, namely, the transmission device, CCD camera, camera fixing device, and control device.

The core device of the CCD camera used in the image acquisition system was the Linea LA-GC-02K05B color line scan camera from Teledyne DALSA. The specific parameters of this device are shown in Table 1. The camera adopts high-sensitivity complementary metal–oxide–semiconductor (CMOS) technology, and the line frequency can reach up to 26 KHz, with high acquisition speed, low noise, high single-line resolution, and high sensitivity. To reduce the influence of light conditions on the acquisition effect during the data acquisition process, ensuring uniform illumination and minimizing the light reflection in the data acquisition area are necessary to improve the imaging quality of the acquired image. Therefore, the linear light source LCOL-300-25 was selected to meet the requirement of uniform illumination in the single-line area required by the linear CCD camera. When collecting data, the scanning frequency is set to 1280 lines for processing once, and after collecting data on the front of each sheet, a 2048 × 18,000 pixel image of a solid wood board with a depth of 8 bits can be obtained.

### 2.2. Preprocessing Images

**Figure 2:**
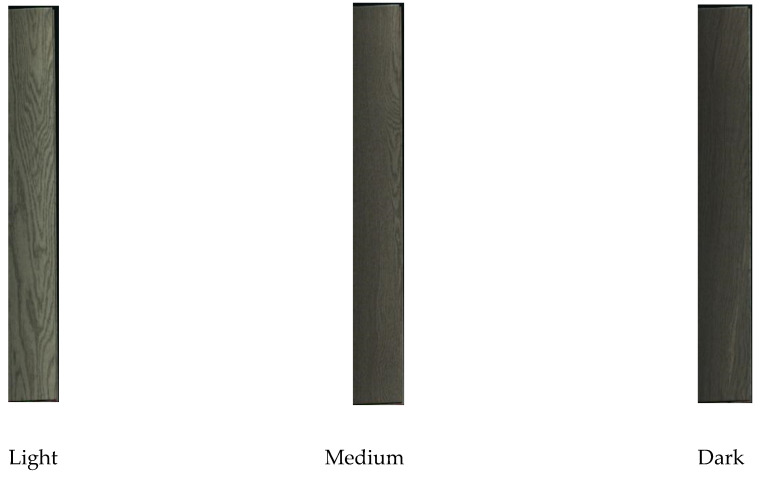
Solid wood floors in three colors.

The solid wood flooring enterprises provided 36 pieces of flooring of each color, making a total of 108 pieces. Example of each color of flooring is shown in Figure 2. After scanning each piece of flooring, the initial dataset was obtained. The distribution of the initial dataset is shown in Table 2.

After graying the three-channel color image in the BMP format collected by the camera, morphological operations, such as corrosion and expansion, were performed. The canny operator was used to obtain the boundary and extract the solid wood flooring image from the black background to reduce the impact of the background. The obtained result is shown in Figure 1. Then, the solid wood floor image, which was removed from the background, was converted into three color spaces. The original image of the three-channel RGB (Red, Green, Blue) was converted into HSV (Hue, Saturation, Value). The laboratory color space, which is based on an international standard for color measurement established by the Commission International Eclairage (CIE) in 1931 and improved in 1976, was used to extract the color features.

The general description methods for color features include color histograms, color moments, color aggregation vectors, and color correlation graphs. This article selects the use of low-order color moments (e.g., first-order and second-order color moments) and the peak of the color histogram as the color features to describe the solid wood flooring. The first-order color moment $\mu_{i}$ and the second-order color moment $\sigma_{i}$ are mathematically defined as follows:

(1) $$\mu_{i}=\frac{1}{N}\overset{N}{\underset{j=1}{\sum }}p_{i,j}$$

(2) $$\sigma_{i}={\left(\frac{1}{N}\overset{N}{\underset{j=1}{\sum }}{\left(p_{i,j}-\mu_{i}\right)}^{2}\right)}^{\frac{1}{2}}$$

where $p_{i,j}$ is the pixel value at the image coordinate, and its actual meaning represents the pixel mean and variance of a picture.

**Figure 3:**
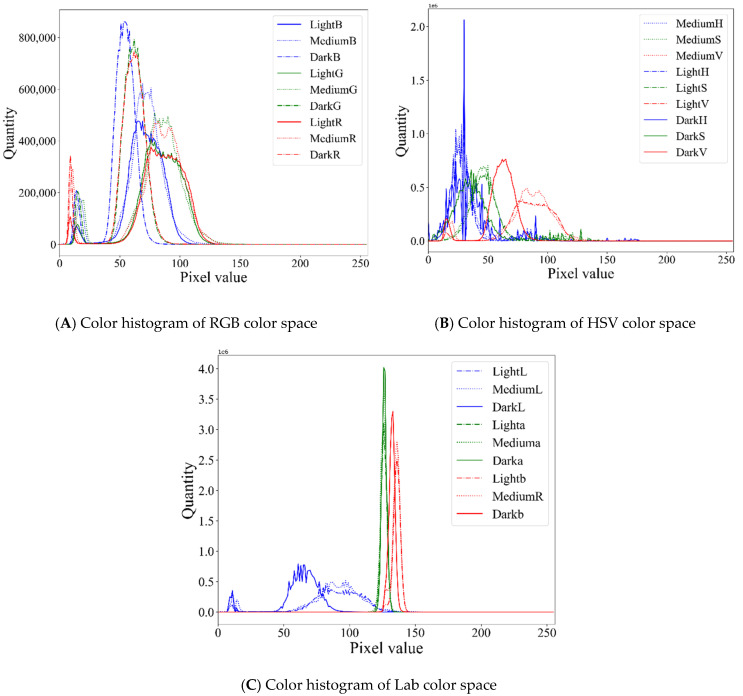
Color histogram of three wood colors in the (**A**) RGB, (**B**) HSV, and (**C**) Lab spaces.

The average value of wood image pixels can be used as a better feature to describe the overall color distribution of wood images. The variance describes the uniformity of the image distribution in the color domain. The color of common wood images will not change greatly. Thus, the second-order color moments can stand for the texture feature in some degree. Generally, the judgment of which color grade solid wood flooring belongs to is often determined by its background color rather than the color of its texture. The background color is often the color with the largest proportion of colors on a solid wood floor. The color histogram of three types of wood board images is shown in Figure 3. The peaks of the color histograms of different colors of solid wood flooring are quite different. Therefore, the peak of the color histogram was selected to represent the background color of the solid wood flooring as a feature of solid wood flooring color classification. Finally, it was planned to use the currently extracted 27 color features to classify the color of the solid wood flooring.

### 2.3. Color Classification Algorithm for Solid Wood Flooring

Common classification models include SVM, multilayer perceptron (MLP), extreme gradient boosting (XGBoost), and deep learning methods. MLP is a forward-structured neural network, and the hidden nodes of the network need to be determined by themselves. The classic SVM algorithm usually performs two classification operations. If multi-classification is performed, then it will increase the training time and computational cost. XGBoost is derived from the gradient boosting framework, but it is more efficient. The algorithm can be calculated in parallel and is effective for sparse data. The processing and memory usage optimization are 10 times faster than the existing gradient boost implementation.

Extreme gradient boosting (XGBoost) [18,19,20] is a tree ensemble model. For a given n of data with m features, the sum of K trees is used as the prediction result.

(3) $$\hat{y_{i}}=\phi \left(x_{i}\right)=\overset{K}{\underset{k=1}{\sum }}f_{k}\left(x_{i}\right),\;f_{k}\in ℱ$$

where $ℱ=\left\{f\left(x\right)=w_{q\left(x\right)}\right\}\left(q:{\mathbb{R}}^{m}\to T,w\in {\mathbb{R}}^{T}\right)$ represents the space of the regression tree, T represents the number of leaves of the tree, each $f_{k}\left(x\right)$ corresponds to the tree structure q and the weight of the leaf $w,\;x_{i}$ represents the $i^{th}$ sample, and $y_{i}$ represents the $i^{th}$ category label.

Establish the loss function:

(4) $$ℒ\left(\phi \right)=\sum_{i}l\left(\hat{y_{i}},y_{i}\right)+\sum_{k}\Omega \left(f_{k}\right),$$

where

(5) $$\Omega \left(f\right)=\gamma T+\frac{1}{2}\lambda {∥w∥}^{2},$$

where l represents the difference between $\hat{y_{i}}$ and $y_{i}$, and Ω is the penalty function on the complexity of the model. The optimization parameter of ℒ(ϕ) is a model, which cannot be optimized by traditional methods. Therefore, the method of addition is used during training, and $f_{t}$ is added in the t round to minimize the following goals:

(6) $${ℒ}^{\left(t\right)}=\overset{n}{\underset{i=1}{\sum }}l\left(y_{i},{\hat{y}}_{i}^{\left(t-1\right)}+f_{t}\left(x_{i}\right)\right)+\Omega \left(f_{t}\right)$$

Carry out the Taylor expansion of the objective function, take the first three terms, and ignore the infinitesimal terms, and obtain

(7) $${ℒ}^{\left(t\right)}≃\sum_{i}^{n}\left[l\left(y_{i},{\hat{y}}^{\left(t-1\right)}\right)+g_{i}f_{t}\left(x_{i}\right)+\frac{1}{2}h_{i}f_{t}^{2}\left(x_{i}\right)\right]+\Omega \left(f_{t}\right),$$

when $g_{i}=\partial_{{\hat{y}}^{\left(t-1\right)}}l\left(y_{i},{\hat{y}}^{\left(t-1\right)}\right),\;h_{i}=\;\partial_{{\hat{y}}^{\left(t-1\right)}}^{2}l\left(y_{i},{\hat{y}}^{\left(t-1\right)}\right)$.

After solving the objective function, the optimal leaf node score is obtained,

(8) $$w_{j}^{*}=\frac{\sum_{i\in I_{j}}g_{i}}{\sum_{i\in I_{j}}h_{i}+\lambda }.$$

Substitute the leaf node scores into the objective function and obtain

(9) $${\tilde{ℒ}}^{\left(t\right)}\left(q\right)=-\frac{1}{2}\overset{T}{\underset{j=1}{\sum }}\frac{{\left(\sum i\in I_{j}g_{i}\right)}^{2}}{\sum_{i\in I_{j}}h_{i}+\lambda }+\gamma T.$$

Under normal circumstances, enumerating all and selecting the best possible tree structures are impossible. Thus, a greedy algorithm is used instead: starting from a single leaf node that is iteratively split to add nodes to the tree, $I_{L},I_{R}$ are the set of left and right nodes after splitting. Then, the loss function after node segmentation is

(10) $${ℒ}_{split}=\frac{1}{2}\left[\frac{{\left(\sum_{i\in I_{L}}g_{i}\right)}^{2}}{\sum_{i\in I_{L}}h_{i}+\lambda }+\frac{{\left(\sum_{i\in I_{R}}g_{i}\right)}^{2}}{\sum_{i\in I_{R}}h_{i}+\lambda }-\frac{{\left(\sum_{i\in I}g_{i}\right)}^{2}}{\sum_{i\in I}h_{i}+\lambda }\right]-\lambda .$$

The tree learning model needs to find the optimal segmentation point. The basic precise greedy algorithm can be used to enumerate all possible partitions of all features to find the optimal segmentation point. When the amount of data is large, an approximate algorithm can be used. This can be used to obtain the percentile, propose n candidate segmentation points, and then map the sample to the two corresponding adjacent segmentation points to form a block, and the objective function can be transformed into

(11) $${\tilde{ℒ}}^{\left(t\right)}=\overset{n}{\underset{i=1}{\sum }}\frac{1}{2}h_{i}{\left(f_{t}\left(x_{i}\right)-\frac{g_{i}}{h_{i}}\right)}^{2}+\Omega \left(f_{t}\right)+constant.$$

**Figure 4:**
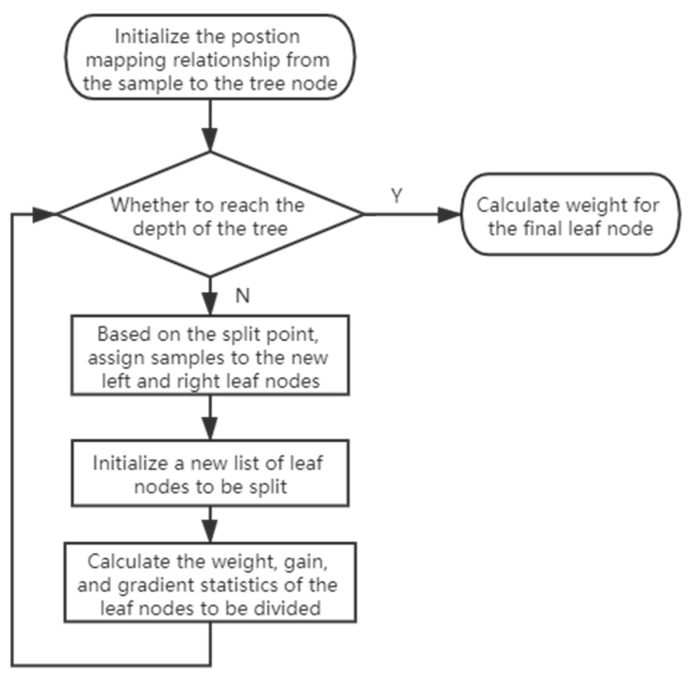
XGBoost algorithm flow.

The algorithm flow chart is shown in Figure 4:

As shown in Figure 4, the XGBoost algorithm, at first, initializes the mapping relationship between the sample and position of the tree node, then it initializes the list of leaf nodes, and after that, it calculates the weight, gain, and gradient of the leaf nodes. If the depth of the tree is reached, it calculates the weight for the final leaf node. If not, it assigns samples to the new left and right leaf nodes based on the split point, then it initializes a new list of leaf nodes to be split and calculates the weight, gain, and gradient of the leaf nodes.

## 3. Results

We extracted the first-order color moment, the second-order color moment, and the peak of the color histogram from the nine color channels of R, G, B, L, a, b, H, S, and V of the solid wood flooring image. Subsequently, XGBoost was used for classification. The XGBoost parameters are presented as follows: the learning rate was 0.001, the number of trees was 1000, the depth of the tree was 3, the minimum weight of the leaf node was 0.1, the penalty term coefficient was 0.2, and Softmax was used as the loss function.

**Figure 5:**
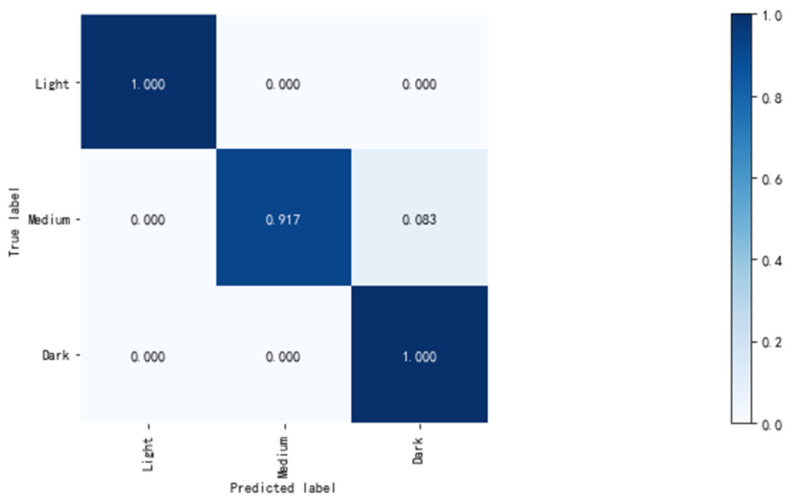
XGBoost classification confusion matrix.

**Figure 6:**
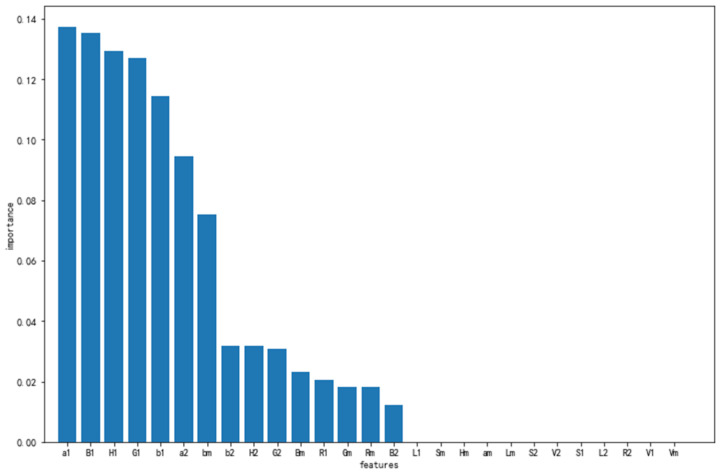
Importance of color features to the classification result ranking diagram.

All the codes of the wood color classification were written in Python, using the machine learning package scikit-learn, which includes the API for XGBoost. The deep learning network used in this paper is the deep learning framework PyTorch to define the network calculation graph. The software, hardware, and compilation environment configuration of this experiment is detailed in Table 3:

The 108 solid wood flooring images obtained by scanning were divided into test and training sets according to 1:3. The accuracy rate of the test set was 97.22%. The confusion matrix of the test results is shown in Figure 5:

The classification accuracy of dark and light colors is high and can be fully recognized, whereas the division of medium colors is fuzzier, which is consistent with the human senses. The reason is that the color classification boundaries of light and medium colors are more obvious. The classification boundary between medium and dark color is blurred, which is related to the color classification standard provided by the manufacturer. Thus, being confused between medium color and dark color is inevitable. The color feature importance value of each of the solid wood flooring images in the XGBoost classification model is calculated, and the feature importance map is sorted. In Figure 6, labels 1, 2, and m on the *x*-axis in the graph represent the first-order moment, second-order moment, and peak value of the color histogram of the corresponding channel. For example, a1 represents the first-order moment of channel “a” in the Lab color space, a2 represents the second-order moment of channel “a” in the Lab color space, and am represents the peak value of the color histogram of channel “a” in the Lab color space.

The first-order moment of the L, S, and V channels; second-order moment of the S, V, L, and R channels; and the color histogram peak value of the S, H, a, L, and V channels have no impact on the classification result. The importance score of the S and V channels is zero, indicating that the saturation value has no effect on the color classification of solid wood flooring. It also indicates the robustness of the model under different lighting conditions. The first-order moments of the a, B, H, G, and b channels have a great influence on the final classification effect. The reason is that the laboratory color space is a color model established on the basis of the human perception of color. The decorativeness of solid wood flooring often depends on human senses, and the color tone of the solid wood flooring is mainly yellowish [21], thereby reflecting the importance of the B and G channels. Table 4 can be obtained by feature screening over a defined threshold:

Table 4 shows that after the first-order moments of the L, S, and V channels; the second-order moments of the S, V, L, and R channels; and the color histogram peaks of the S, H, a, L, and V channels are removed, the color classification can still be performed with 97.22% accuracy, although from Table 4, at least three feature values, namely, the color first moments of the a, B, and H channels, can be used for color classification. However, for the stability of the model, the color feature whose contribution is not zero is still used for prediction. After re-extracting 15 valid features, a new XGBoost model was re-established. After the new classification model was retrained, the classification accuracy rate of 97.22% was still obtained in the test set.

## 4. Discussion

At present, with the development of image classification technology, deep learning technology is used in many image processing works. Common deep learning networks include vgg16, Resnet, and Densenet. The process of extracting eigenvalues can be skipped by using deep learning technology. The structure of some deep learning networks is shown in Table 5 and Table 6.

In order to improve the training effect of deep learning, the left–right symmetry, top–down symmetry, and diagonal symmetry of the removed background image were used to obtain the expanded dataset. The expanded dataset is divided into the test set and training set according to 1:3. The distribution of the dataset is shown in Table 7.

**Figure 7:**
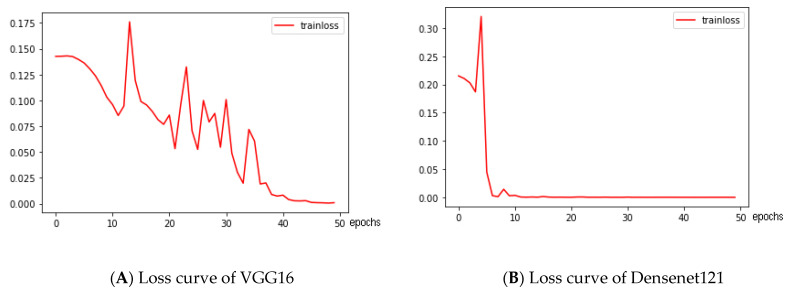
Loss curves of deep learning models.

The deep learning model was trained under the following parameters: the loss function is cross-entropy, the optimizer is SGD, the learning rate is 0.01, the batch size is 16, and the number of epochs is 50. In order to speed up the model training, the pretrained model on the Imagenet dataset is used for transfer learning. The loss curves of deep learning models is shown in Figure 7.

The comparison of using color features for prediction and convolutional neural network effects is presented as follows. To use XGBoost, we need to extract image features first, then train the classification model for the extracted features, so the time used for the two tasks is calculated respectively.

Table 8 shows that the classification method based on feature values can obtain higher classification accuracy with less training time. The classification accuracy of the deep learning classification model increases with depth. The time used by the XGBoost classification model is much shorter than that of the convolutional neural network method. Given that XGBoost uses regularization terms to prevent overfitting and considers the case where the training data are sparse values, the impact of missing data on the model can be ignored. In addition, the model can be obtained faster and is more suitable for training small samples because the XGBoost model is faster than the deep learning training models, such as VGGNet16 and Densenet121. Therefore, online learning can be realized, and the daily influence of external factors on the color classification of solid wood flooring can be reduced during actual production.

In this study, the image resolution obtained by the CCD line scan camera is relatively large and the data volume of the whole solid wood flooring image is large. Therefore, the feature extraction of the solid wood flooring image takes a long time and consumes a large memory space, thereby affecting the speed of online detection to a certain extent. Therefore, designing an efficient feature extraction algorithm is necessary.

## 5. Conclusions

In this study, the first-order color moments, the second-order moments of the nine color channels, and the peak value of the color histograms of R, G, B, L, a, b, H, S, and V of the solid wood flooring image were extracted and then classified using the XGBoost model to complete the coloring of the target solid wood flooring. After dividing the 108 pictures obtained by scanning into test and training sets according to a 1:3 ratio, the average accuracy rate of the 27 test set pictures after testing was 97.2%. The classification accuracy for dark and light colors can reach 100%, the classification accuracy for medium colors was 91.7%, and the average classification time for a single solid wood floorboard was 51 ms. After comparing the XGBoost classification model with the classification effects of different deep learning networks, such as VGG16 and Densenet121, XGBoost has great advantages in terms of model training and model classification speed. In particular, the training speed required to obtain the classification model is several orders of magnitude faster, as well as the classification accuracy, than that of the deep learning model.

## Figures and Tables

**Table 1:** Specific parameters of the CCD camera.

Parameter Name	Parameter	Parameter Name	Parameter
Model	Linea LA-GC-02K05B	Spectrum	Color
Sensor Technology	CMOS	Operating Temperature	0 °C–65 °C
Resolution	2048 × 2	Pixel Depth	8-bit
Pixel Size	7.04 × 7.04 µm	Horizontal Frequency	Up to 26 KHz

**Table 2:** Distribution of the initial dataset.

Color	Light	Medium	Dark
Quantity	36	36	36

**Table 3:** Software and hardware environment configuration table.

Name	Parameter
System	Windows 10 × 64
CPU	Inter Core i7-10700@2.90GHz
GPU	Nvidia GeForce GTX 2060 SUPER(8G)
Environment configuration	PyCharm + Pytorch1.2.0 + Python3.7.7 + skcikitlearn0.23.1 Cuda10.0 + cudnn7.6 + tensorboardX2.1.0

**Table 4:** Classification accuracy threshold obtained by using different color feature numbers.

Threshold	Feature Number	Accuracy (%)
0	27	97.22
0.01211	15	97.22
0.018069	14	97.22
0.01828	13	97.22
0.020515	12	97.22
0.023294	11	97.22
0.03068	10	97.22
0.031688	9	97.22
0.031797	8	97.22
0.075192	7	97.22
0.094652	6	97.22
0.114261	5	97.22
0.127119	4	97.22
0.129506	3	97.22
0.135398	2	94.44
0.137441	1	69.44

**Table 5:** VGG16 construction.

Layers	Output Size	Densnet121
Convolution	224 × 224	3 × 3 conv-64
		3 × 3 conv-64
Pooling	112 × 112	max pool
Convolution	112 × 112	3 × 3 conv-128
		3 × 3 conv-128
Pooling	56 × 56	max pool
Convolution	56 × 56	3 × 3 conv-256
		3 × 3 conv-256
		3 × 3 conv-256
Pooling	28 × 28	max pool
Convolution	28 × 28	3 × 3 conv-512
		3 × 3 conv-512
		3 × 3 conv-512
Pooling	14 × 14	max pool
Convolution	14 × 14	3 × 3 conv-512
		3 × 3 conv-512
		3 × 3 conv-512
Classification Layer	1 × 1	max pool
		4096D fully connected
		4096D fully connected
		1000D fully connected, softmax

**Table 6:** Densenet121 construction.

Layers	Output Size	Densnet121
Convolution	112 × 112	7 × 7conv, stride 2
Pooling	56 × 56	3 × 3 max pool, stride 2
Dense Block (1)	56 × 56	×6
Transition Layer (1)	56 × 56	1 × 1 conv
	28 × 28	2 × 2 average pool, stride 2
Dense Block (2)	28 × 28	×12
Transition Layer (2)	28 × 28	1 × 1 conv
	14 × 14	2 × 2 average pool, stride 2
Dense Block (3)	14 × 14	× 24
Transition Layer (3)	14 × 14	1 × 1 conv
	7 × 7	2 × 2 average pool, stride 2
Dense Block (4)	7 × 7	×16
Classification Layer	1 × 1	7 × 7 global average pool
		1000D fully connected, softmax

**Table 7:** Distribution of the expanded dataset.

	Test Set	Training Set
Dark	36	108
Medium	36	108
Light	36	108

**Table 8:** Effect of different classification methods for the same test set.

Classification Method	Classification Accuracy (%)	Training Time (s)	Classification Time (s)
VGG16	0.77	1002.667	2.43
Densenet121	0.94	1069.11	2.11
XGBoost	0.97	Image Feature Extraction Time: 4.08	Image Feature Extraction Time: 1.044
		Training Time: 0.19	Training Time: 0.09

## Data Availability

The data are not publicly available due to the company requirements.
